# Natural selection mediated by seasonal time constraints increases the alignment between evolvability and developmental plasticity

**DOI:** 10.1111/evo.14147

**Published:** 2021-01-06

**Authors:** Frank Johansson, Phillip C. Watts, Szymon Sniegula, David Berger

**Affiliations:** ^1^ Department of Ecology and Genetics, Animal Ecology Uppsala University Uppsala 752 36 Sweden; ^2^ Department of Biological and Environmental Science University of Jyväskylä Jyväskylä 40014 Finland; ^3^ Department of Ecosystem Conservation, Institute of Nature Conservation Polish Academy of Sciences Krakow 31–120 Poland

**Keywords:** Adaptation, developmental bias, G‐matrix, genetic constraints, latitude, life history, phenotypic plasticity, time constraints

## Abstract

Phenotypic plasticity can either hinder or promote adaptation to novel environments. Recent studies that have quantified alignments between plasticity, genetic variation, and divergence propose that such alignments may reflect constraints that bias future evolutionary trajectories. Here, we emphasize that such alignments may themselves be a result of natural selection and do not necessarily indicate constraints on adaptation. We estimated developmental plasticity and broad sense genetic covariance matrices (**G**) among damselfly populations situated along a latitudinal gradient in Europe. Damselflies were reared at photoperiod treatments that simulated the seasonal time constraints experienced at northern (strong constraints) and southern (relaxed constraints) latitudes. This allowed us to partition the effects of (1) latitude, (2) photoperiod, and (3) environmental novelty on **G** and its putative alignment with adaptive plasticity and divergence. Environmental novelty and latitude did not affect **G**, but photoperiod did. Photoperiod increased evolvability in the direction of observed adaptive divergence and developmental plasticity when **G** was assessed under strong seasonal time constraints at northern (relative to southern) photoperiod. Because selection and adaptation under time constraints is well understood in *Lestes* damselflies, our results suggest that natural selection can shape the alignment between divergence, plasticity, and evolvability.

Whether phenotypic plasticity hinders or facilitates adaptation in new environments has been debated, and recently interest in this issue has intensified due to the increasing need to understand species’ responses to global environmental change (Whitlock 1996; Price et al. 2003; West‐Eberhard 2003; Ghalambor et al. 2007; Lande 2009; Chevin et al. 2010; Walters et al. 2012; Levis and Pfennig 2016; Uller et al. 2018; Levis and Pfennig 2019; Noble et al. 2019). One argument is that, because phenotypic plasticity can rapidly generate phenotypes that are better matched to novel environmental settings, plasticity can allow organisms to survive the initial stages of change, leaving an opportunity for subsequent genetic adaptation through natural selection (Waddington 1953; West‐Eberhard 2003; Lande 2009; Levis and Pfennig 2016). This mechanism, in turn, might have important implications for rates of genetic diversification (Gomez‐Mestre and Buchholz 2006; Pfennig et al. 2010; Susoy et al. 2015). The potential for plasticity to facilitate adaptation in new environments should thus depend on whether plastic responses move the phenotype closer to the new adaptive peak and whether there is additive genetic variation allowing traits to respond in the direction of selection. Adaptive evolution is therefore predicted to be further facilitated, if both genetic variation and phenotypic plasticity are aligned with the direction of multivariate selection generated by the environmental change (Lande 2009, but see Whitlock 1996; Walters et al. 2012).

Against this background, a prominent debate has surfaced about causation in the evolutionary process and why such an alignment might be expected in the first place (e.g., Gerhart and Kirschner 2007; Laland et al. 2015; Houle et al. 2017; McGlothlin et al. 2018; Uller et al. 2018; Svensson and Berger 2019; Jiang and Zhang 2020). Classic interpretations of an alignment between genetic variation and divergence typically invoke genetic constraints, suggesting that adaptation is constrained to occur along dimensions for which there is additive genetic variation (Lande 1979; Schluter 1996). Similarly, developmental plasticity is argued to play a leading role in evolution by biasing the evolutionary trajectory along certain developmental pathways predetermined by persistent selection during an organism's evolutionary history (West‐Eberhard 2003): a phenomenon often referred to as “developmental bias” (Uller et al. 2018). Indeed, the common influence of developmental bias during the course of evolution might be inferred from the fact that development tends to produce certain phenotypes more often than others (Uller et al. 2018). However, it is also possible that an alignment between developmental plasticity, genetic variation, and divergence could be created over relatively short time scales by contemporary natural selection shaping all three levels of biological variation simultaneously (Cheverud 1984; Schluter 1996; Houle et al. 2017; Noble et al. 2019; Svensson and Berger 2019). Hence, such an alignment does not need to reflect constraints on adaptation. Here, correlational selection can canalize the effects of both genetic (de novo mutations or segregating variation) and environmental perturbation on phenotypic expression, causing favorable trait combinations to occur and be selected more frequently than expected by chance. Indeed, recent theory suggests that such effects can accumulate within a few hundred generations (Draghi and Whitlock 2012; Jones et al. 2014). Alignments between developmental plasticity, genetic variation, and divergence may thus have dual causality. Determining the extent to which such alignments are a consequence of genetic/developmental constraints or the adaptive outcomes of contemporary natural selection is important for predicting evolutionary potential: the former alternative implies a limited capacity for adaptation to novel selection pressures, in contrast to the latter alternative that implies potential for rapid adaptive responses.

Because new environments often confer some change in selection on coadapted trait complexes, it is not obvious that alignments between ancestral plasticity and genetic variation will increase evolvability upon environmental change. Increased evolvability could nevertheless occur if exposure to an environmental change releases “cryptic genetic variation” (Paaby and Rockman 2014), such that genetic variation increases overall in new environments. A recent meta‐analysis by Noble et al. (2019) found that phenotypic plasticity was aligned with the amount of genetic (co)variance within and between traits, but whether genetic variation was quantified in the ancestral or a novel environment had no overall effect on the alignment or the total amount of expressed variation. These results were discussed with regard to the “plasticity first hypothesis,” stating that plasticity takes the lead in adaptive evolution (Levis and Pfennig 2016; Radersma et al. 2020). However, identifying unambiguous support for the idea that plasticity is an evolutionary driver, and not itself a product of selection, is difficult. In addition to information on plasticity and genetic (co)variation in the studied traits, knowledge of trait divergence and multivariate phenotypic selection is ultimately necessary (Levis and Pfennig 2016; Noble et al. 2019).

Correlated genetic and plastic responses to selection need to be studied using a multivariate approach, which can be achieved by examining the additive genetic variance and covariance between traits as summarized in a G‐matrix (Arnold et al. 2008). Quantifying **G** across alternative environments can provide information about how well plastic responses and additive genetic (co)variances are aligned. Making this type of analysis among multiple populations, locally adapted to the alternative environments for which multivariate selection is understood, offers the opportunity to test the prediction that the alignment between divergence, plasticity, and **G** is related to the differences in natural selection imposed by the respective environments. Here, we applied this approach in the damselfly *Lestes sponsa* (Odonata: Zygoptera), along a latitudinal gradient in Europe, to explore how multivariate selection mediated via seasonal time constraints affects the alignment between developmental plasticity, **G**, and divergence.

Seasonal environments impose strong selection for phenotypic plasticity in growth and development (Bradshaw and Holzapfel 2001, 2004; Sniegula et al. 2016, 2018), where photoperiod often is used as a reliable cue for tracking time and inducing adaptive plasticity (Tauber et al. 1986). At northern latitudes, a short growth season imposes strong time constraints on development and selects for a fixed strategy determining age and size at maturity, with fast development and a small size at emergence, and lack of standing genetic variation in growth rate (Rowe and Ludwig 1991; Abrams et al. 1996; Hoffmann and Parsons 1997; Dmitriew 2011). Support for these predictions has been found in several time‐constrained insect populations (Bradshaw and Holzapfel 2001, 2004; Blanckenhorn and Demont 2004; Nygren et al. 2008; Berger and Gotthard 2008; Gotthard 2008). The relaxed time constraints experienced at more southern latitudes, on the other hand, facilitate growth to a larger body size, a more flexible age at maturity, and leave room for alternative foraging strategies (e.g., the trade‐off between juvenile growth and mortality risk mediated by foraging effort: Werner and Gilliam 1984).

In the damselfly *L. sponsa*, the genetic and plastic responses of juvenile growth to seasonal time constraints (cued by photoperiod) follow these general patterns and are likely adaptive, with evidence for local adaptation in the use of photoperiodic cues (Sniegula and Johansson 2017; Sniegula et al. 2017). We manipulated photoperiod to simulate time constraints and induce changes in multivariate selection on growth and development in populations of *L. sponsa* sampled from a latitudinal gradient in Europe. We compared northern and southern populations for several life history and morphological traits to quantify (1) phenotypic plasticity in response to photoperiod, (2) population divergence in mean trait values, and (3) the broad‐sense genetic covariance matrix (**G**) measured at both photoperiods. This experimental design allowed us to assess effects of latitude (southern vs. northern origin) and photoperiod (northern photoperiod that induces strong time constraints vs. a more southern photoperiod with its more relaxed time constraints) on **G** and its potential alignment with developmental plasticity and adaptive divergence. We performed three main tests aimed at determining evolutionary causality:

- We tested for the presence of an alignment between **G**, plasticity, and divergence, which would suggest a putative role for developmental bias and constraints in our study system. However, evidence against such a constraint includes previous work showing that plasticity in response to photoperiod and divergence along latitude in *L. sponsa* both reflect adaptive responses to seasonal time constraints (Sniegula and Johansson 2010; Sniegula et al. 2017).
- Because an alignment between plasticity and **G** does not per se indicate constraints on adaptation, we tested if seasonal time constraints (an effect of photoperiod) affected the alignment between plasticity and **G**. As multivariate selection on the measured life history phenotypes is known to be induced by seasonal time constraints, an effect of photoperiod on the alignment would thus suggest natural selection as its causal driver. An effect of photoperiod on the alignment between **G**, plasticity, and divergence is, however, not informative as to whether natural selection shaped the alignment over long periods of time during the evolutionary history of *L. sponsa*, or whether the alignment has been shaped over a more recent and shorter time scale.
- Our third test was therefore to examine whether differences in contemporary selection among our northern and southern populations had shaped the alignment. We did this by exploring if the effect of photoperiod on the alignment differed between the two types of populations. Because northern and southern populations differ in their recent evolutionary history, signified by stronger seasonal time constraints and observed concomitant responses to photoperiod in northern populations (Sniegula and Johansson. 2017; Sniegula et al. 2017), a stronger effect of photoperiod treatment on the potential alignment is predicted in northern populations. We note here that southern populations need to use photoperiodic cues to time their development and have experienced time constraints during their evolutionary history (e.g., during the latest ice age in central Europe), and indeed, also show plasticity in response to perceived time constraints (Sniegula and Johansson. 2017; Sniegula et al. 2017). Hence, the southern populations can also be expected to show effects of photoperiod on the alignment under the “contemporary selection hypothesis” (but less so than the northern populations).

## Methods

### STUDY SYSTEM

Our study organism was the obligatory univoltine damselfly *Lestes sponsa* (Hansemann 1823). In Europe, *L. sponsa*’s latitudinal distribution extends from northern Spain (Boudot and Kalkman 2015) to northern Sweden (Artportalen 2019). After a winter diapause, *L. sponsa* hatch in spring and the aquatic juvenile stage lasts for 2‐3 months after which the terrestrial adult stage has a lifespan of about a month (Corbet 1956). All growth occurs during the larval stage, but mass increase can occur in adults (Hyeun‐Ji and Johansson 2016). Because *L. sponsa* is time constrained, development time and size at emergence impact fitness (De Block and Stoks 2005). In addition, morphological traits such as wing morphology and thorax size have consequences for fitness components such as mating and survival (Swillen et al. 2009; Outomuro et al. 2016). Natural selection has apparently shaped adaptive phenotypic plasticity in this species. For example, across a latitudinal gradient covering central to northern Europe, individuals grown in a southern photoperiod take on average 25% longer to develop into adults when compared with those in a northern photoperiod, and this is an insufficient development time for the short season in the north (Sniegula and Johansson 2010). Also, in the congeneric *L. viridis*, time constraints result in lower mating success (De Block and Stoks 2005), suggesting that plasticity that accelerates development time until metamorphosis should be adaptive. Moreover, northern populations of *L. sponsa* are probably experiencing directional selection for faster growth and egg development time because these traits have lower additive genetic variance in the north (Sniegula et al. 2016).

### SAMPLING AND REARING OF POPULATIONS

In 2018, we collected eggs from *L. sponsa* females that had been sampled from three latitudes in Europe: northern Sweden (northern: 66°N; *n* = 34 females), central Sweden (central: 59°N; *n* = 36), and north‐western Poland (southern: 54°N; *n* = 38) on 1 August, 23‐27 July and 8‐14 August, and 29‐30 July, respectively. Although Poland is not in southern Europe, we refer to these populations as north, central, and south (i.e., in a latitudinal context) for simplicity. We have no data about the level of genetic divergence among these populations, but development time, growth rate, and additive genetic variance for growth rate and egg development time differ significantly between populations of *L. sponsa* that span the latitudinal range used in our study (Sniegula et al. 2016). Because of the last ice age, the northern population probably split from the southern ones about 5000 years ago. We sampled seven populations: three, two, and two sites from the north, central, and south regions, respectively. All females were collected as mating pairs to form full‐sib families. Mated females were allowed to lay eggs in individual cups (see Supplementary Information 1 for details on animal husbandry). Eggs were allowed to overwinter in their native condition, in one of three climate chambers representing the temperature and photoperiod (derived lake model FLake [Lake Model FLake 2009]), of the southern, central, and northern sampling locations. We selected a combination of source individuals and thermo‐photoperiods to give five experimental groups: (1) southern (temperature‐photoperiod) and native (genetic origin of eggs), (2) central (temperature‐photoperiod) and native (genetic origin of eggs), (3) northern (temperature‐photoperiod) and native (genetic origin of eggs), (4) southern (temperature‐photoperiod) and northern (genetic origin of eggs), and (5) northern (temperature‐photoperiod) and southern (genetic origin of eggs). Note that because photoperiod is the main developmental cue in *L. sponsa* (Norling 2018), and because degree days (i.e., the total temperature sum over development) until emergence does not differ much between latitudes (Supplementary Information 2), we refer to photoperiod as the main environmental difference between latitudinal sample locations throughout the manuscript. On 20 November 2018, we initiated spring conditions for the eggs (i.e., water temperature >10°C, the threshold temperature for spring hatching in *L. sponsa*; Corbet 1956), and then simulated weekly changes of spring and summer temperatures and photoperiods until the last larva had emerged. Hence, eggs and larvae received continuous changes (weekly) in temperature and photoperiod during development and growth, simulating the conditions at south, central, and northern latitudes. Six larvae per female and treatment (*n* = 1156 larvae) were reared individually (see, data archiving) until emergence, after which individuals were weighed and preserved in 95% ethanol. We measured eight traits for three to six larvae from each clutch: larval development time between hatching and emergence, adult body mass at emergence, head width, thorax length, thorax width, abdomen length, tibia length (third leg on the right side), and wing length (posterior right wing). Exact landmarks, used to characterize the phenotype (see Supplementary Information 3), were measured from the first larvae that emerged from each clutch, with one clutch of eggs typically consisting of 50‐100 eggs.

### STATISTICS

We assume that offspring within clutches are full‐sibs given apparent sperm precedence in damselflies (Corbet 1999, p. 521), although some clutches in *L. sponsa* may be half‐sibs (Johansson et al. 2020). Maternal effects on larval life history traits are low in *L. sponsa* (<1% in a study by Sniegula et al. 2016), suggesting that our quantitative genetic analysis of full‐sibs will mostly describe broad sense genetic variation, but does not exclude the presence of maternal environmental effects.

All traits were first mean‐standardized (Hansen and Houle 2008). Fitting multivariate response models that included all eight original traits proved unfeasible. As tibia length, abdomen length, thorax length and width, and head width were positively correlated, we therefore calculated a composite trait that described variation in metric size: metric size = (tibiaL × abdomenL × thoraxL × thoraxW × headW)^1/5^. Hence, we analyzed four traits: (1) metric size, (2) (linearized) body mass^1/3^, (3) wing length, and (4) development time. As all traits were approximately normally distributed, we assumed Gaussian response variables in all analyses.

We first tested whether there was significant latitudinal variation in each trait among the seven populations when reared at their respective native photoperiods. We applied mixed models using restricted maximum likelihood (REML) estimation and the Kenward‐Rodgers correction for the denominator degrees of freedom using lme4 (Bates et al. 2015) and car (Fox and Weisberg 2019) packages in R version 3.6.1 (R Core Team 2019), with latitude and sex as fixed effects and sample population as a random effect, making sure that each population serves as an independent observation of the effect of latitude on trait variation (see Supplementary Information 4 for model specification and output).

Next, we estimated whether there was (1) significant genetic differentiation in the four traits, (2) an effect of photoperiod treatment on trait expression (i.e., plasticity), and (3) significant genetic differentiation in the use and interpretation of photoperiod to cue development (i.e., a latitude:photoperiod treatment interaction). We used mixed models for each trait excluding the two populations from central latitude (that were reared only at their own native photoperiod). The data from the central latitude populations were thus used only to examine if there was a latitudinal cline in expressed life history traits: populations from two latitudes will (almost) always differ in one way or another. These models thus compared the populations from northern and southern latitude that had been reared in the common garden experiment including both the northern (long) and southern (short) photoperiods, and the changes in temperature. Latitude, photoperiod treatment, and sex were included as fully crossed fixed effects, and population crossed with photoperiod treatment was included as a random effect to ensure the correct level of replication for the effects of latitude and the latitude:photoperiod interaction. The family identity crossed with offspring sex was included as an additional random effect (model specification and output in Supplementary Information 5).

To compare G‐matrices, we used multivariate response models including the four traits in a Bayesian mixed modeling framework incorporating Markov Chain Monte Carlo simulations using the MCMCglmm package (Hadfield 2010) in R (model specification in Supplementary Information 6). We blocked out effects of population, sex, and photoperiod by adding these variables as fully crossed fixed effects. To estimate **G**, for each combination of latitude (northern and southern latitude) and photoperiod treatment (northern and southern day length), we fitted family identity as a latitude‐ and photoperiod‐specific random effect, with broad sense genetic (co)variances approximated as twice the (co)variance among full‐sibs. To retrieve corresponding trait heritabilities, the residual covariance matrix was fitted for latitude and photoperiod treatment. There was little indication of sex‐specific genetic variance in any of our single‐trait analyses (Supplementary Information 5). Likewise, there was no statistical support for population‐specific genetic covariance structure between populations within each latitude (results not shown). Hence, we estimated the four G‐matrices by averaging across sexes and populations within each latitude. We used weak and unbiased priors for the covariance matrices as recommended by Hadfield (2019) (Supplementary Information 6). There was much greater variation in development time compared to the morphological traits and all calculations and comparisons (described further below) were thus based on variance standardized matrices. In our models, genetic variance is expressed as units of phenotypic standard deviations and, therefore, twice the family variance in trait expression corresponds to (broad sense) trait heritability. We ran models for 550,000 iterations, discarding the first 50,000 iterations (as burn‐in) and stored every 500th run, resulting in 1000 uncorrelated posterior estimates of **G** for each latitude:photoperiod combination. These posterior estimates were used to calculate posterior modes (the most probable value) and Bayesian 95% credible intervals for all matrix parameters as well as to calculate *P*‐values for all matrix comparisons (see below).

First we compared trait‐specific genetic variation and heritability across the four latitude:photoperiod combinations. We then compared overall genetic variation by summing the variance across all four traits (i.e., the trace of **G**). We then inspected the orientation of **G** by performing eigen‐analysis of **G** and its corresponding genetic correlation matrix, **R** (the latter removing the influence of differences in overall genetic variance across traits). We explored how the first eigen‐vector, explaining most of the variation in **G** and **R** (**G_max_** and **R_max_** from here on), loaded on the four original traits, giving a representation of the orientation of the major axis of genetic variation and the genetic correlation structure across the four latitude:photoperiod combinations. We also explored how much of the total variance in **G** and **R** was explained by **G_max_** and **R_max_**, giving a representation of matrix dimensionality. We quantified (dis)similarity in the orientation of **G_max_** and **R_max_**, respectively, across the four groups by calculating pairwise vector correlations:

(1) $$\;r_{g1,g2}=\frac{g1g2}{\left|g1\right|\left|g2\right|}\;,$$

where *g*1 and *g*2 are **G_max_** or **R_max_** for group 1 and group 2, |*g*1| and |*g*2| are the corresponding vector norms, and $r_{g1,g2}$ is the correlation between the vectors, ranging from 0 (maximum dissimilarity; vectors are completely orthogonal in multivariate trait space) to 1 (perfect similarity; vectors are identical).

Testing for statistical significance of correlations for **G_max_** and **R_max_** directly is problematic as the estimated vector correlations are bounded between 0 and 1. Therefore, to formally test for differences in the orientation of **G**, and simultaneously test the hypothesis that **G** is aligned with adaptive plasticity, we compared the alignment between **G** and the direction of multivariate developmental plasticity between the four latitude:photoperiod combinations via two complementary approaches (see also Berger et al. 2013; Lind et al. 2015). First, we calculated the alignment between **G_max_** and the multivariate vector of developmental plasticity for all four latitude:photoperiod combinations separately:

(2) $$\;\theta_{G_{\max},\mathrm{DP}}=\cos^{-1}\;\frac{G_{\max}\mathrm{DP}}{\left|G_{\max}\right|\left|\mathrm{DP}\right|},$$

where *DP* is the multivariate vector of developmental plasticity, given by the changes in trait means in response to photoperiod treatment, and $\theta_{G_{\max},\mathrm{DP}}$ is the angle between **G_max_** and developmental plasticity in multivariate trait space, ranging from 90 (maximum dissimilarity; vectors are completely orthogonal in multivariate trait space) to 0 (perfect similarity; vectors are identical). Second, following Hansen and Houle (2008), we quantified evolvability (***e***) of **G** along the vector of multivariate developmental plasticity

(3) $$e_{\mathrm{DP}}=\frac{DP^{\prime }G_{\max}\mathrm{DP}}{{\left|\mathrm{DP}\right|}^{2}},\;$$

where *DP’* is the transpose of *DP*. ***e_DP_*** thus gives the expected response to selection along the plasticity vector for a unit strength of selection acting in the same multivariate direction as plasticity. We calculated posterior modes and 95% credible limits for all these metrics based on the Bayesian posterior estimates. We performed two‐sided hypotheses tests for the effect of latitude (eq. 4a), photoperiod (eq. 4b), and for differences in the effect of photoperiod between geographic origins (eq. 4c), by computing effect sizes for both alignment and evolvability based on the 1000 stored posterior estimates:

(4a) $$\;\mathrm{Effec}t_{\mathrm{origin}}=N_{S}\;+N_{N}-S_{S}-S_{N},$$

(4b) $$\;\mathrm{Effec}t_{\mathrm{photo}}=N_{S}\;+\;S_{S}-N_{N}-S_{N},\;\mathrm{and}$$

(4c) $$\mathrm{Effec}t_{\mathrm{origin}:\mathrm{photo}}=\left[N_{S}\;-N_{N}\right]-\;\left[S_{S}-S_{N}\right],$$

where *N_N_* and *N_S_* are estimates from northern populations raised at northern and southern photoperiod, and *S_N_* and *S_S_* are estimates from southern populations raised at northern and southern photoperiod. Bayesian *P*‐values were calculated by counting the fraction of stored iterations where the calculated effect overlapped 0 and then multiplying this fraction by a factor of 2 (to retrieve the two‐sided *P*‐value). As the alignment between **G_max_** and the vector of developmental plasticity essentially is a fraction (eq. 2), posterior estimates were logit transformed to achieve normality and to relativize computed differences prior to calculating 95% credible intervals and *P*‐values.

To test whether evolvability in the direction of multivariate plasticity was greater than expected by chance for each latitude:photoperiod combination, we computed evolvability for random matrices drawn from a multivariate normal distribution using the mvrnorm function in R. Matching random matrices were created for each of our 1000 stored posterior estimates of the empirically derived matrices. To do this, we first calculated the summed genetic variance for the four traits of the estimated matrix (i.e., the trace of **G**) for each stored iteration. We then created a random unstructured G‐matrix by sampling the same number of full‐sib families as in our original data (*N_N_* = 39, *N_S_* = 33, *S_N_* = 42, and *S_S_* = 43) from a multivariate normal distribution with the same amount of total genetic variance as for the estimated matrix, but assuming homogeneous trait variances and covariances = 0. We then calculated pairwise evolvabilities for the empirical matrix and the resulting unstructured random matrix for each of the 1000 stored iterations (eq. 3) and used these to calculate two‐sided Bayesian *P*‐values for each latitude:photoperiod combination as described above.

## Results

### LATITUDINAL VARIATION, DEVELOPMENTAL PLASTICITY, AND GENETIC DIFFERENTIATION IN TRAIT MEANS

**Figure 1:**
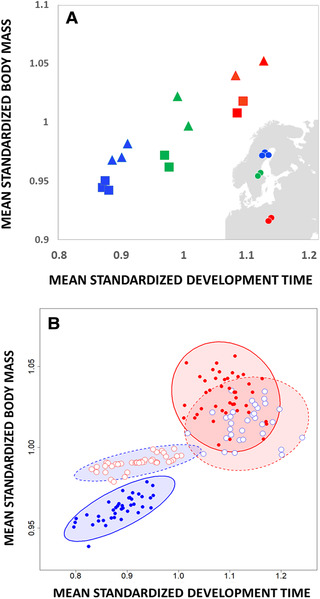
(A) significant latitudinal variation in body mass and development time among seven populations reared at their native photoperiod length. Male values are represented by squares and female values are represented by triangles. Dots on the map show sample location of the populations studied. Standard errors are <0.01 and not shown. (B) geographic variation and developmental plasticity in body mass and development time. Shown are breeding values estimated from the Bayesian mixed model run on mean‐standardized traits (model specification in Supplementary Information 6). Breeding values for southern and northern populations are shown as red and blue points, respectively. Breeding values from populations raised at their native and nonnative photoperiod are filled and open points, respectively, and surrounded by 95% confidence ellipses drawn with full and broken lines, respectively. The northern photoperiod caused a significant shift in the orientation of **G** in the direction of multivariate developmental plasticity, seen in populations of both northern and southern origin. Northern populations have evolved stronger plasticity in response to the photoperiod cue relative to southern populations.

There was significant latitudinal variation in all four traits when larvae were raised at their native photoperiod (*P* < 0.01 for all traits, Supplementary Information 4), with southern populations generally entering metamorphosis at an older age and larger size (Fig. 1A). In accordance, we identified significant differentiation in the three morphological traits in the common garden experiment (all *P* < 0.05, Supplementary Information 5), with southern populations being larger. All four traits showed strong plastic responses to photoperiod treatment, with a northern photoperiod generally rendering the faster development and smaller body size that is expected under perceived time constraints (all *P* < 0.01, Supplementary Information 5). There was a significant latitude‐by‐photoperiod interaction for development time (*P* = 0.009, *F*_1:2.48_ = 56.7) and a marginally nonsignificant interaction for body mass (*P* = 0.066, *F*_1:2.52_ = 9.70), signifying genetic divergence along the direction of multivariate plasticity and local adaptation in the use of photoperiodic cues to program development (Supplementary Information 5). Consistent with the putative effect of strong time constraints, the northern populations, evolving under stronger seasonal time constraints at northern latitude, showed a stronger response to photoperiod (Fig. 1B).

### G‐MATRIX COMPARISONS

When comparing the variance‐standardized broad sense genetic (co)variance matrix, **G**, across latitudes (northern vs. southern Europe) and photoperiod treatments (northern vs. southern day length), there was a tendency for an increase in broad sense genetic variance and heritability for development time in southern populations raised at the novel northern photoperiod. However, there was no concomitant increase in the northern populations when they were reared at a southern photoperiod (Fig. 1B). Moreover, there was no tendency for genetic variance to increase in novel photoperiod when considering all four traits, either separately or when summing the total genetic variance over all traits (i.e., the trace of **G**), suggesting little evidence for revealed “cryptic” genetic variation in a novel photoperiod (summary in Table S7A).

When exploring the orientation of **G** and **R**, we found pronounced effects of photoperiod treatment on the trait loadings of **G_max_** and **R_max_** (summary in Table S7B). Here, growth in a northern photoperiod, signaling the strong time constraints experienced at northern latitude, led to positive correlations between development time and the three morphological traits, as expected in a scenario with a trade‐off between age and size at maturity and multivariate stabilizing selection favoring a fixed growth maximization strategy under seasonal time constraints. In the southern photoperiod, however, giving the perception of relaxed time constraints as experienced at southern latitude, development time and the three morphological traits were uncorrelated. The change in **G_max_** and **R_max_** caused genetic covariance structure to be more similar across populations from different latitudes raised at the same photoperiod, than for the same population raised at different photoperiods (Fig. 1B and Table 1). We quantified this (dis)similarity by estimating vector correlations of **G_max_** and **R_max_**, respectively, across latitudes and photoperiod treatments (Table S7B). This analysis showed that, indeed, vector correlations between matrices from populations reared at the same photoperiod were close to unity, whereas the novelty of the photoperiod treatment or latitude had relatively little effect on similarity of **G_max_** or **R_max_** (Table S7B).

**Table 1:** Genetic correlation matrix for each origin (blue frame: northern, red frame: southern) and photoperiod (blue background: northern/long photoperiod, red background: southern/short photoperiod). Negative (positive) correlations are coded in dark (light) colors

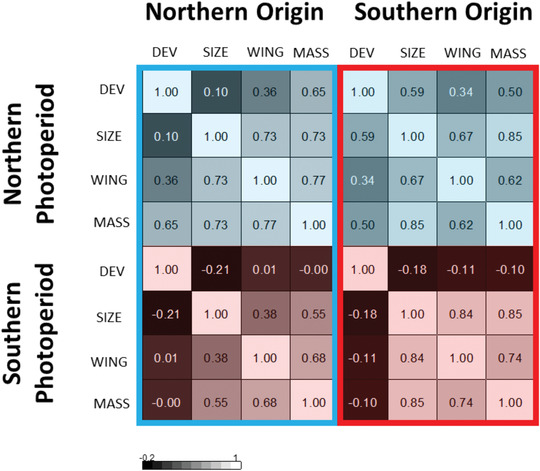

### ALIGNMENT BETWEEN G AND MULTIVARIATE DEVELOPMENTAL PLASTICITY

**Figure 2:**
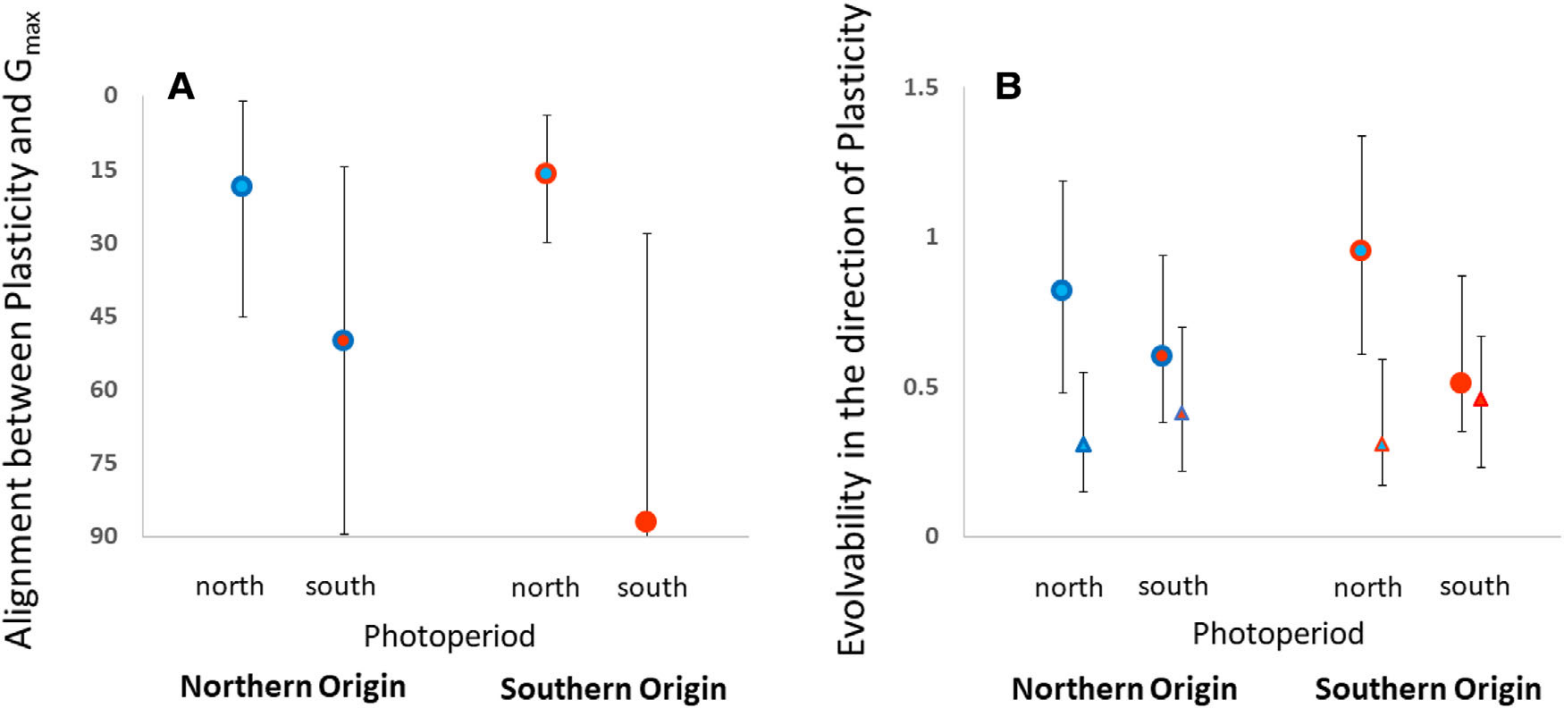
(A) The alignment between multivariate developmental plasticity and the major axis of genetic variation, **G_max_**, given for northern (blue borders) and southern (red borders) populations raised at northern (blue background) or southern (red background) photoperiod. An angle = 0 indicates complete alignment and an angle = 90 indicates that the directions of multivariate plasticity and **G_max_** are orthogonal. (B) Standardized evolvability in the direction of multivariate developmental plasticity. Circles show estimates based on empirical data and triangles show null expectations based on simulated unstructured G‐matrices with homogeneous variances and covariances set to 0. Given are Bayesian posterior modes and 95% credible intervals. See main text for further details.

Latitudinal origin had no significant effect on alignment or evolvability (*P*_MCMC_ > 0.6). However, the photoperiod treatment had a large influence on the alignment between **G_max_** and plasticity; development in the northern photoperiod (increased time constraints) caused genetic covariances to align with the direction of multivariate developmental plasticity, and this increase in alignment (relative to southern photoperiod and relaxed time constraints) was significant (*P*_MCMC_ = 0.048; Fig. 2A). Similarly, northern photoperiod tended to increase evolvability in the direction of plasticity (relative to the southern photoperiod), although the effect was not statistically significant (*P*_MCMC_ = 0.10; Fig. 2B). Moreover, comparing evolvability for the estimated G‐matrices and unstructured (random) matrices containing the same amount of total genetic variation showed that evolvability in the direction of multivariate plasticity was greater than expected by chance for the northern photoperiod (northern latitude: *P*_MCMC_ = 0.01, southern latitude: *P*_MCMC_ < 0.001), but not for the southern photoperiod (northern latitude: *P*_MCMC_ = 0.26, southern latitude: *P*_MCMC_ = 0.27) (Fig. 2B). We found no evidence suggesting that the effect of photoperiod differed between northern and southern populations (both origin:photoperiod interactions: *P*_MCMC_ > 0.8), although we note that the power of our analysis would only pick up very pronounced differences as being statistically significant. In summary, developmental plasticity and evolvability were aligned only when **G** was measured at the photoperiod that signals strong multivariate selection via seasonal time constraints. However, as the alignment in northern and southern populations showed a similar response to photoperiod, we did not find evidence to suggest that any relatively recent change in selection at the different latitudes has affected the alignment.

## Discussion

### ALIGNMENT CAUSED BY SELECTION

Our analyses of populations of the damselfly *L. sponsa* uncovered that **G** was aligned with the direction of (multivariate) adaptive developmental plasticity and divergence in response to seasonal time constraints, only when **G** was estimated in the photoperiodic conditions that signal seasonal time stress. This suggests that the observed alignment is not a result of pure constraints, but rather implies that changes in natural selection imposed by seasonality have been instrumental in shaping the observed alignment between **G** and phenotypic plasticity. Indeed, insects that experience seasonal time constraints pay large fitness costs of suboptimal growth strategies due to intensified costs of postponing age at maturity and are therefore expected to show (i) fixed growth maximization strategies (Fig. 1B) (Rowe and Ludwig 1991; Abrams et al. 1996) and (ii) adaptive developmental plasticity in response to photoperiod (e.g., Bradshaw and Holzapfel 2001; Gotthard 2008), as shown previously in *Lestes* (Johansson and Rowe 1999; De Block and Stoks 2005; Sniegula et al. 2014). A scenario where selection shapes plasticity in both trait means and their genetic (co)variances will facilitate adaptation when **G** and plasticity are aligned also with the selection pressures imposed in the new environment so that (1) expressed phenotypes are moved closer toward the new trait optima, and (2) there is abundant genetic variation for the phenotypes in the direction of increased fitness, leaving opportunity for subsequent genetic assimilation (Lande 2009; Chevin et al. 2010, but see Whitlock 1996; Walters et al. 2012). However, if selection in the new environment is truly novel, then ancestral plasticity may be maladaptive and selected against (Ghalambor et al. 2015) even if it was originally derived from past selection. The extent to which such “misaligned” ancestral plasticity will limit future adaptation should depend on how fast the new selection pressures can reshape genetic (co)variances and phenotypic plasticity.

### PAST OR CONTEMPORARY SELECTION

Theory suggests that alignments between phenotypic plasticity and genetic variation can, rather rapidly, develop if both levels of biological variation are shaped by correlational selection on underlying interacting genetic loci (Arnold et al. 2008; Draghi and Whitlock 2012; Jones et al. 2014). However, our comparison of northern populations (currently experiencing strong seasonal time constraints) and southern populations (currently experiencing more relaxed selection from seasonality) found no support for a difference in alignment between **G**, plasticity, and divergence. We note, however, that statistical power to identify more nuanced differences in the alignment was limited in our study, as often is the case for quantitative genetic designs. The result suggests that past selection, rather than contemporary selection following the evolutionary split of our northern and southern populations during the last ice age, has caused the observed alignment. Our study thus implies that the observed plasticity in trait means and in **G** itself is ancestral, but leaves open the question of how much developmental constraints may bias future adaptive potential to seasonality under climate change. Even though we did find that the northern populations have evolved a stronger plastic response in trait means to photoperiod cues than southern populations, southern populations also show strong responses to photoperiod (Fig. 1B). This may suggest that southern populations either currently also experience nontrivial selection to interpret cues from long day lengths or experienced such selection pressures in the not so distant past and have since then been under relaxed selection. Both these explanations are compatible with the observed effect of photoperiod on the alignment in both northern and southern populations and leave open the possibility that strong directional selection under climate change could result in a rapid change in the alignment between plasticity and **G**. Admittedly, photoperiod was different in the ancestral populations of *L. sponsa* during the last ice age when this species withdrew to the south. However, photoperiod would still have signaled time constraints because photoperiod varies over the growth season and affects southern *Lestes* populations (Johansson and Rowe 1999; De Block and Stoks 2005; Sniegula et al. 2014).

### CRYPTIC GENETIC VARIATION

A population that enters a novel environment may exhibit an increase in genetic variation via release of “cryptic” genetic variation (McGuigan Sgro 2009; Paaby and Rockman 2014). An obvious scenario for release of cryptic genetic variation is when a population expands its range margin into new environments (Nadeau and Urban 2019). As a release of cryptic genetic variation reveals novel phenotypes upon which selection can act, this process has been proposed to have the potential to facilitate adaptation to novel environments (Hayden et al. 2011; McGuigan et al. 2011; Zheng et al. 2019). In our study, raising the populations at the nonnative photoperiod should be interpreted as novel conditions because both types of populations have not encountered these photoperiodic schemes for thousands of generations. Our 2 × 2 study design allowed us to separate the effects on **G** stemming from novelty of the photoperiod cue (i.e., southern populations raised at northern photoperiod, and vice versa) from those stemming from photoperiod per se. However, we found no general support for a release of cryptic variation in the studied life history traits, nor an effect of environmental novelty on **G** and its alignment with plasticity. In a meta‐analysis by Wood and Brodie (2015), environmental novelty did not affect differences in **G** between environments significantly. Similarly, in the meta‐analysis by Noble et al. (2019), the alignment between **G** and plasticity was unaffected (on average) by a change in the environment. Wood and Brodie (2015) concluded that their nonsignificant results might be caused by the absence of an objective metric of environmental novelty. Our study supports this conclusion and sheds further light on both these meta‐analyses as our 2 × 2 design allowed us to partition the effects of environmental novelty and photoperiod to show that only the latter had strong effects on **G** and its alignment with plasticity. As for many similar studies quantifying selection and genetic variation in ancestral and novel environments, one reason for why there was no excess of “cryptic” genetic variation released at the novel photoperiods in our study could be that selection in nature is still acting on the genes underlying responses to photoperiod, or has done so in the recent evolutionary past (see discussion above), even though the applied experimental day lengths are not experienced currently in the natural populations.

## Conclusions

The evolutionary causality of alignments between phenotypic plasticity and additive genetic variance is of fundamental importance for realized adaptive potentials in changing environments. Here, we have shown that the alignment between **G** and developmental plasticity in phenotypes under multivariate selection from seasonal time constraints increases in an environment manipulated to increase such time constraints. This suggests that the type and strength of multivariate selection perceived by the organism can reshape alignments between plasticity and **G**. Although several studies have found alignments between plasticity and **G** (Noble et al. 2019), the causal explanation for such alignments cannot be provided by correlational studies (Houle et al. 2017; McGlothlin et al. 2018; Uller et al. 2018; Rohner et al. 2019) and our study suggest that this association can be the result of past changes in the selective environment rather than hard constraints on developmental trajectories. Our study did, however, not find any evidence suggesting that more recent changes in selection occurring between northern and southern populations have affected the alignment. Interestingly, the meta‐analysis by Noble et al. (2019) showed that the alignment between plasticity and **G** can strongly depend on the type of environmental change studied. Hence, more studies are needed to explore how past and present environmental selection shapes changes in plasticity, **G**, and their alignment, preferably using simultaneous information on multivariate selection and (adaptive and nonadaptive) genetic divergence in the studied phenotypes.

## AUTHOR CONTRIBUTIONS

FJ, PCW, and DB designed the study. FJ and SS collected the data. DB analyzed the data. FJ, PCW, SS, and DB wrote this article.

## CONFLICT OF INTEREST

The authors declare no conflict of interest.

Associate Editor: M. R. Walsh

Handling Editor: D. W. Hall

## Supporting information


**Table S1**: Photoperiod and temperature used for each latitude treatment in the experiment simulating the progress of the season at each latitude.
**Figure S1**: Morphological measurements on *Lestes sponsa*: (a) head width, (b) tibia length, (c) wing length, (d) abdomen length, (e) thorax width, (f) thorax width.
**Table S7a**: Bayesian posterior estimates of mean‐standardized broad sense genetic variance and heritability for each of the four traits.
**Table S7b**: Best Bayesian posterior estimates of **Gmax** and **Rmax**.

## Data Availability

Data are deposited on Dryad (https://doi.org/10.5061/dryad.jsxksn07z).
