# Supplementary material for: Natural selection mediated by seasonal time constraints increases the alignment between evolvability and developmental plasticity
Source: Evolution. 2021 Jan 6;75(2):464–75. doi: 10.1111/evo.14147 (PMC7986058; doi:10.1111/evo.14147)
Supplement: Supplementary file 1 — Table S1: Photoperiod and temperature used for each latitude treatment in the experiment simulating the progress of the season at each latitude. Figure S1: Morphological measurements on Lestes sponsa: (a) head width, (b) tibia length, (c) wing length, (d) abdomen length, (e) thorax width, (f) thorax width. Table S7a: Bayesian posterior estimates of mean‐standardized broad sense genetic variance and heritability for each of the four traits. Table S7b: Best Bayesian posterior estimates of Gmax and Rmax. [file EVO-75-464-s001.pdf]

Supporting Information, Johansson et al.

Natural selection mediated by seasonal time constraints increases the alignment between evolvability and developmental plasticity.

Supplementary 1

After catching the mated females were individually put in a small plastic cups with a lid. Each cup had a moist filter paper aligned at the inside of cup wherein the female oviposited the eggs after 1-3 days. The eggs were then transported to a laboratory. Upon arrival to the laboratory the eggs were introduced in the climate chambers where they were overwintered and reared in three climate chambers, one with southern thermo-photoperiod, one with central thermo-photoperiod and one with northern thermo-photoperiod. Half of the southern eggs and half of the northern eggs were reared in the southern climate chamber and the other halves were reared in the northern climate chamber. All the central eggs were reared in the central climate chamber. This resulted in five groups; Southern/native, Central/native, Northern/native, Southern/northern, and Northern/southern (the groups are named after the pattern latitude/photoperiod).

The climate chambers had temperature and photoperiods that simulating the time of the year (late summer) at their respective region of collection (south, central and north). The temperatures used during the experiment were derived from Flake model (Lake Model Flake, 2009). Simulation of winter conditions, temperature 7 °C and photoperiod L-D 00:00-24:00, started on 4, 5 and 7 September 2018 for southern, central and northern latitude populations, respectively, and ended on 20 November 2018 for the three latitude populations. We initiated spring conditions dates when water temperature exceeds 10 °C at each sampling latitude because a threshold temperature for spring hatching in L. sponsa is 10 °C (Corbet, 1956). On 20 November 2018, we programmed southern region conditions for 10.9 °C, L-D 14:56-09:04, which corresponded to 12 April 2019, central region conditions for 10.6 °C and L-D 17:04-06:56, which corresponded to 26 April 2019, and northern region conditions for 10.6 °C and L-D 24:00-00:00, which corresponded to 24 May 2019. From these dates, we started simulating weekly changes of spring and summer temperatures and photoperiods until the end of the experiment, that is, when the last larva emerged.

During spring conditions, all eggs hatched after about ten days. The larvae were reared individually in 200 ml round plastic cups filled with 100 ml of aged tap water and fed twice a day (morning and afternoon feeding) between Monday-Friday and once a day between Saturday-Sunday with laboratory cultured Artemia nauplii, (mean 215 nauplii/fed, SE = 7, N = 10). When entering into one prior final instar, the larval feeding was supplemented with three standard-size life midge larvae every Monday, Wednesday and Friday. The day after emergence, individuals were weighed and then preserved in ethanol. Six offspring from each female was raised. However, only 1-2 individuals per female were later measured. This resulted in a total of1156 larvae at the start of the experiment. The following

traits were measured on emerged adults: larval development time between hatching and emergence, adult body mass at emergence, head width, thorax length, thorax width, abdomen length, tibia length and wing length. For the tibia length, the tibia from the third leg on the right side was measured. For the wing length, the posterior right wings were measured. For exact measurement points, see Figure S1 in supplement.

In our statistical analysis we assume that the offspring are full-sibs. First, sperm precedence, the fraction of female eggs fertilized by the last male to copulate with a female is variable in damselflies but rarely less than 95% (Corbet 1999, page 521). But an unpublished study suggests that this percentage might be somewhat variable (Johansson et al. 2020) in L. sponsa. Maternal effects on larval life history traits was found to be less than 1% in a previous study (Sniegula et al 2016).

Supplementary 2

Table S1. Photoperiod and temperature used for each latitude treatment in the experiment simulating the progress of the season at each latitude. The first column is the date used simulating the actual photoperiod and temperature. L denotes light of and D denotes light on, and temp is the temperature used.

POLAND: 53.651 N, 16.343 E			
2019	L	D	temp. + 3
19-04-12	14:56	09:04	10,9
19-04-19	15:28	08:32	12,6
19-04-26	15:59	08:01	14,3
19-05-03	16:30	07:30	17,1
19-05-10	17:00	07:00	18,2
19-05-17	17:28	06:32	18,2
19-05-24	17:54	06:06	19,2
19-05-31	18:16	05:44	17,2
19-06-07	18:34	05:26	21,1
19-06-14	18:46	05:14	20,6
19-06-21	18:51	05:09	21,5
19-06-28	18:48	05:12	22
19-07-05	18:39	05:21	22,2
19-07-12	18:24	05:36	22,8
19-07-19	18:03	05:57	22,8
19-07-26	17:38	06:22	22,9
19-08-02	17:12	06:48	23,7
19-08-09	16:42	07:18	23,1
19-08-16	16:12	07:48	21,8
19-08-23	15:41	08:19	21,6
19-08-30	15:11	08:49	20,7
19-09-06	14:39	09:21	19,1
19-09-13	14:08	09:52	18,8
19-09-20	13:38	10:22	16,8
19-09-27	13:08	10:52	15,6
19-10-04	12:38	11:22	14,7
19-10-11	12:09	11:51	13,1

SWEDEN: 59.84 N, 17.66 E			
2019	L	D	temp. + 3
19-04-26	17:04	06:56	10,6
19-05-03	17:48	06:12	13,7
19-05-10	18:32	05:28	14,9
19-05-17	19:17	04:43	16,2
19-05-24	20:01	03:59	17,1
19-05-31	20:45	03:15	17,9
19-06-07	21:25	02:35	18,9
19-06-14	21:57	02:03	19,7
19-06-21	22:13	01:47	20,3
19-06-28	22:05	01:55	20,7
19-07-05	21:38	02:22	21,9
19-07-12	21:01	02:59	23,1
19-07-19	20:18	03:42	23,1
19-07-26	19:35	04:25	22,5
19-08-02	18:49	05:11	22,8
19-08-09	18:05	05:55	22,2
19-08-16	17:22	06:38	21,6
19-08-23	16:40	07:20	20,5
19-08-30	15:58	08:02	19,1
19-09-06	15:16	08:44	17,7
19-09-13	14:37	09:23	16,8
19-09-20	13:58	10:02	14,9
19-09-27	13:19	10:41	14
19-10-04	12:41	11:19	12,5
19-10-11	12:05	11:55	11,1
19-10-18	11:29	12:31	9,1
19-10-25	10:53	13:07	7,8

SWEDEN: 65.701 N, 21.872 E			
2019	L	D	temp. + 3
19-05-24	24:00:00	00:00	10,6
19-05-31	24:00:00	00:00	15
19-06-07	24:00:00	00:00	17,3
19-06-14	24:00:00	00:00	18,1
19-06-21	24:00:00	00:00	18,6
19-06-28	24:00:00	00:00	20,3
19-07-05	24:00:00	00:00	21,5
19-07-12	24:00:00	00:00	21,2
19-07-19	24:00:00	00:00	21,2
19-07-26	24:00:00	00:00	20,7
19-08-02	22:51	01:08	20,6
19-08-09	20:40	03:20	20,2
19-08-16	19:19	04:41	19
19-08-23	18:10	05:50	17,5
19-08-30	17:08	06:52	16,2
19-09-06	16:12	07:48	14,9
19-09-13	15:17	08:43	13,5
19-09-20	14:26	09:34	11,2
19-09-27	13:35	10:25	10
19-10-04	12:47	11:13	7,9
19-10-11	12:00	12:00	6,3
19-10-18	11:13	12:47	5,1
19-10-25	10:27	13:33	3,7
19-11-01	09:44	14:16	3,1

Supplementary 3

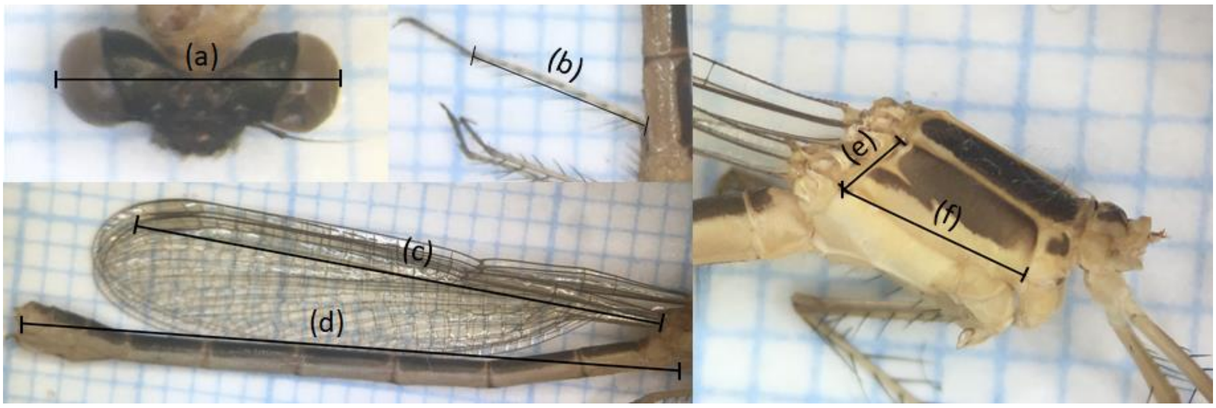

Figure S1. Morphological measurements on *Lestes sponsa*: (a) head width, (b) tibia length, (c) wing length, (d) abdomen length, (e) thorax width, (f) thorax width

SUPPLEMENTARY 4

Test of latitudinal variation in the four traits

# DEVELOPMENT TIME

```
lmer(devtimeM ~ sex+latitude + (0+sex|female)+
(1|pop),na.action=na.exclude,data=cryp2)->moddev

Random effects:
 Groups   Name        Variance  Std.Dev. Corr
 female   sexf        1.967e-03 0.044349
          sexm        1.630e-03 0.040369 0.94
 pop      (Intercept) 1.492e-06 0.001222
 Residual             2.321e-03 0.048178
Number of obs: 615, groups:  female, 118; pop, 7

Fixed effects:
              Estimate Std. Error t value
(Intercept)  2.0320376  0.0518554  39.187
sexm        -0.0181672  0.0046649  -3.894
latitude    -0.0172658  0.0008674 -19.905

Analysis of Deviance Table (Type II Wald F tests with Kenward-Roger df)

Response: devtimeM
              F Df  Df.res    Pr(>F)
sex       15.03  1 100.573 0.0001888 ***
latitude 367.25  1   3.999  4.38e-05 ***
```

#BODY MASS

```
lmer(log(massM) ~ sex+latitude + (0+sex|female)+ (1|pop)
,na.action=na.exclude,data=cryp2)->modmass

Random effects:
 Groups   Name        Variance Std.Dev. Corr
 female   sexf        0.002342 0.04839
          sexm        0.003353 0.05790  0.60
 pop      (Intercept) 0.000374 0.01934
 Residual             0.006498 0.08061
Number of obs: 578, groups:  female, 118; pop, 7

Fixed effects:
             Estimate Std. Error t value
(Intercept)  1.075832   0.111826   9.621
sexm        -0.104269   0.008941 -11.662
latitude    -0.017637   0.001855  -9.510


Analysis of Deviance Table (Type II Wald F tests with Kenward-Roger df)
```

```
Response: log(massM)
                F Df  Df.res    Pr(>F)
sex      134.837  1 101.188 < 2.2e-16 ***
latitude  88.229  1   4.681 0.0003269 ***
```

#WING LENGTH

```
lmer(WingM ~ sex+latitude + (0+sex|female)+ (1|pop),
na.action=na.exclude,data=cryp2)->modwing

Random effects:
 Groups   Name        Variance  Std.Dev. Corr
 female   sexf        0.0002662 0.01631
          sexm        0.0003456 0.01859  0.65
 pop      (Intercept) 0.0000000 0.00000
 Residual             0.0009647 0.03106
Number of obs: 554, groups:  female, 118; pop, 7

Fixed effects:
             Estimate Std. Error t value
(Intercept)  1.3966281  0.0237366   58.84
sexm        -0.0611600  0.0033125  -18.46
latitude    -0.0062830  0.0003968  -15.84

Analysis of Deviance Table (Type II Wald F tests with Kenward-Roger df)

Response: WingM
              F Df  Df.res    Pr(>F)
sex      336.43  1 100.072 < 2.2e-16 ***
latitude 230.97  1   4.053 0.0001001 ***
```

#METRIC SIZE

```
lmer(SIZE ~ sex+latitude + (0+sex|female)+
(1|pop),na.action=na.exclude,data=cryp2)->modSIZE

Random effects:
 Groups   Name        Variance  Std.Dev. Corr
 female   sexf        1.475e-04 0.012146
          sexm        3.315e-04 0.018208 0.90
 pop      (Intercept) 1.377e-05 0.003711
 Residual             8.080e-04 0.028426
Number of obs: 554, groups:  female, 118; pop, 7

Fixed effects:
             Estimate Std. Error t value
(Intercept)  1.3543317  0.0273614  49.498
sexm        -0.0234780  0.0028892  -8.126
latitude    -0.0058231  0.0004553 -12.789

Analysis of Deviance Table (Type II Wald F tests with Kenward-Roger df)

Response: SIZE
              F Df Df.res    Pr(>F)
sex       65.21  1 96.204 1.945e-12 ***
latitude 156.55  1  4.480 0.0001179 ***
```

SUPPLEMENTARY 5

Test of genetic differentiation in the four traits

#DEVELOPMENT TIME

```
lmer(devtimeM ~ sex*region*treat + (0+sex|female)+ (1|pop) +
(1|treat:pop),na.action=na.exclude,data=cryp[cryp$region!="se.c",])->moddev

Random effects:
 Groups    Name        Variance  Std.Dev. Corr
 female    sexf        1.742e-03 0.041737
           sexm        1.527e-03 0.039077 1.00
 treat:pop (Intercept) 2.127e-05 0.004612
 pop       (Intercept) 8.138e-05 0.009021
 Residual              2.976e-03 0.054553
Number of obs: 804, groups:  female, 82; treat:pop, 10; pop, 5

Fixed effects:
                           Estimate Std. Error t value
(Intercept)                 1.098139   0.010743 102.221
sexm                       -0.019864   0.007667  -2.591
regionse.n                  0.039274   0.015279   2.570
treathigh                  -0.176431   0.008564 -20.601
sexm:regionse.n             0.005638   0.011974   0.471
sexm:treathigh              0.015170   0.010820   1.402
regionse.n:treathigh       -0.067391   0.012242  -5.505
sexm:regionse.n:treathigh -0.024879   0.016572  -1.501

Analysis of Deviance Table (Type II Wald F tests with Kenward-Roger df)

Response: devtimeM
                        F Df Df.res    Pr(>F)
sex               13.4473  1  74.47 0.0004577 ***
region             0.0031  1   2.48 0.9599534
treat            1606.6099  1   2.37 0.0002045 ***
sex:region         0.7272  1  77.98 0.3963910
sex:treat          0.3101  1 721.29 0.5778202
region:treat      56.6757  1   2.48 0.0090711 **
sex:region:treat   2.2387  1 725.86 0.1350316
```

#BODY MASS

```
lmer(massM^(1/3) ~ sex*region*treat + (0+sex|female)+ (1|pop)+
(1|treat:pop),na.action=na.exclude,data=cryp[cryp$region!="se.c",])-
>modmass

Random effects:
 Groups    Name        Variance  Std.Dev. Corr
 female    sexf        1.154e-04 0.010744
           sexm        1.547e-04 0.012438 1.00
 treat:pop (Intercept) 9.430e-06 0.003071
 pop       (Intercept) 0.000e+00 0.000000
 Residual              7.764e-04 0.027863
Number of obs: 742, groups:  female, 82; treat:pop, 10; pop, 5

Fixed effects:
                           Estimate Std. Error t value
(Intercept)                 1.044570   0.003715 281.207
sexm                       -0.035094   0.004100  -8.559
regionse.n                 -0.021387   0.005442  -3.930
treathigh                  -0.033930   0.004906  -6.916
sexm:regionse.n             0.015044   0.006338   2.374
sexm:treathigh             -0.005712   0.005774  -0.989
regionse.n:treathigh       -0.017434   0.006875  -2.536
sexm:regionse.n:treathigh -0.002964   0.008727  -0.340

Analysis of Deviance Table (Type II Wald F tests with Kenward-Roger df)

Response: massM^(1/3)
                        F Df Df.res    Pr(>F)
sex              215.7233  1  73.88 < 2.2e-16 ***
region            40.9440  1   2.40  0.014729 *
treat            232.0320  1   2.51  0.001529 **
sex:region         9.3087  1  76.37  0.003136 **
sex:treat          2.5940  1 688.04  0.107730
region:treat       9.6962  1   2.52  0.066298 .
sex:region:treat   0.1141  1 689.65  0.735630
```

#WING LENGTH

```
lmer(WingM ~ sex*region*treat + (0+sex|female)+ (1|pop)+
(1|treat:pop),na.action=na.exclude,data=cryp[cryp$region!="se.c",])-
>modwing

Random effects:
 Groups    Name        Variance  Std.Dev.  Corr
 female    sexf        1.449e-04 1.204e-02
           sexm        2.817e-04 1.678e-02 0.85
 treat:pop (Intercept) 0.000e+00 0.000e+00
 pop       (Intercept) 7.215e-11 8.494e-06
 Residual              1.182e-03 3.438e-02
Number of obs: 705, groups:  female, 82; treat:pop, 10; pop, 5

Fixed effects:
                           Estimate Std. Error t value
(Intercept)                1.061280   0.003673 288.916
sexm                      -0.061635   0.005426 -11.359
regionse.n                -0.039091   0.005722  -6.832
treathigh                 -0.031780   0.004756  -6.682
sexm:regionse.n            0.016428   0.008392   1.957
sexm:treathigh            -0.007690   0.007359  -1.045
regionse.n:treathigh      -0.006330   0.007005  -0.904
sexm:regionse.n:treathigh -0.004863   0.011193  -0.434

Analysis of Deviance Table (Type II Wald F tests with Kenward-Roger df)

Response: WingM
                        F Df Df.res    Pr(>F)
sex              398.7165  1  73.78 < 2.2e-16 ***
region            84.2945  1   2.28  0.007618 **
treat            187.9537  1   2.09  0.004394 **
sex:region         5.3004  1  76.21  0.024052 *
sex:treat          3.0789  1 653.15  0.079785 .
region:treat       2.1331  1   2.36  0.263248
sex:region:treat   0.1861  1 655.63  0.666319
```

#METRIC SIZE

```
lmer(SIZE ~ sex*region*treat + (0+sex|female)+ (1|pop)+
(1|treat:pop),na.action=na.exclude,data=cryp[cryp$region!="se.c",])-
>modSIZE

Random effects:
 Groups    Name        Variance  Std.Dev. Corr
 female    sexf        1.691e-04 0.013004
           sexm        2.397e-04 0.015482 0.99
 treat:pop (Intercept) 0.000e+00 0.000000
 pop       (Intercept) 4.576e-05 0.006764
 Residual              9.130e-04 0.030216
Number of obs: 705, groups:  female, 82; treat:pop, 10; pop, 5

Fixed effects:
                            Estimate Std. Error t value
(Intercept)                1.0420952  0.0058980 176.686
sexm                      -0.0251932  0.0046415  -5.428
regionse.n                -0.0384210  0.0082125  -4.678
treathigh                 -0.0258973  0.0041923  -6.177
sexm:regionse.n           -0.0003611  0.0071905  -0.050
sexm:treathigh            -0.0028792  0.0064776  -0.444
regionse.n:treathigh      -0.0096801  0.0061860  -1.565
sexm:regionse.n:treathigh  0.0110025  0.0098529   1.117

Analysis of Deviance Table (Type II Wald F tests with Kenward-Roger df)

Response: SIZE
                        F Df Df.res    Pr(>F)
sex               94.7078  1  72.67 8.382e-15 ***
region            31.3393  1   2.69  0.015019 *
treat            143.3430  1   2.07  0.006021 **
sex:region         1.1554  1  75.48  0.285849
sex:treat          0.1473  1 648.36  0.701294
region:treat       1.1099  1   2.35  0.388220
sex:region:treat   1.2348  1 650.48  0.266889
```

Supplementary 6

Specification of Bayesian mixed effects model estimating G

```
prior2 = list(

R = list(
R1 = list(V = diag(0.0001, 4), nu = 4),
R2 = list(V = diag(0.0001, 4), nu = 4),
R3 = list(V = diag(0.0001, 4), nu = 4),
R4 = list(V = diag(0.0001, 4), nu = 4)),

G = list(
G1 =  list(V = diag(0.0001, 4), nu = 4, alpha.V=diag(4)*1000, alpha.mu=rep(0,4)),
G2 =  list(V = diag(0.0001, 4), nu = 4, alpha.V=diag(4)*1000, alpha.mu=rep(0,4)),
G3 =  list(V = diag(0.0001, 4), nu = 4, alpha.V=diag(4)*1000, alpha.mu=rep(0,4)),
G4 =  list(V = diag(0.0001, 4), nu = 4, alpha.V=diag(4)*1000, alpha.mu=rep(0,4))
))

modmulti2 <- MCMCglmm(cbind(devtimeM,SIZE,WingM,massM^(1/3)) ~
trait*sex*pop*treat ,

random = ~
us(trait:at.level(region, "se.n"):at.level(treat, "high")):female +
us(trait:at.level(region, "se.n"):at.level(treat, "central")):female +
us(trait:at.level(region, "pl"):at.level(treat, "high")):female +
us(trait:at.level(region, "pl"):at.level(treat, "central")):female ,

rcov = ~
us(trait:at.level(region, "se.n"):at.level(treat, "high")):units +
us(trait:at.level(region, "se.n"):at.level(treat, "central")):units +
us(trait:at.level(region, "pl"):at.level(treat, "high")):units +
us(trait:at.level(region, "pl"):at.level(treat, "central")):units ,
data = cryp3,
family = rep("gaussian",4), prior = prior2, nitt=55000,slice=TRUE,
burnin=5000, thin=50, verbose = T, pr=TRUE)
```

Supplementary 7

G-matrix summaries

Supplementary Table 7a: Bayesian posterior estimates of mean-standardized broad sense genetic variance and heritability for each of the four traits. The trace of each G-matrix (i.e. summed diagonal elements) is given with 95% credible intervals within parentheses. Dimensionality of G and the genetic correlation matrix (R) is given by the percent of total variance explained by the first eigen vector (G_{max} and R_{max}, respectively). For G_{max}, values within parentheses given the corresponding value for variance standardized G-matrix.

Origin	Photoperiod	Genetic Variance				Broad sense heritability				Trace	$\%G_{max}$	$\%R_{max}$
		DEV	SIZE	WING	MASS	DEV	SIZE	WING	MASS			
North	Northern (long)	2.35	0.18	0.20	0.10	0.92	0.30	0.30	0.22	2.9 (1.3-4.3)	81 (57)	80
North	Southern (short)	2.35	0.39	0.002	0.21	1.12	0.62	0.003	0.48	3.0 (2.0-6.2)	83 (51)	74
South	Northern (long)	3.54	0.03	0.29	0.05	1.38	0.06	0.55	0.22	3.9 (2.1-5.3)	90 (64)	82
South	Southern (short)	1.70	0.22	0.20	0.24	0.88	0.40	0.43	0.67	2.3 (1.2-4.2)	76 (59)	90

Supplementary Table 7b: Best Bayesian posterior estimates of G_{max} and R_{max} (i.e. the first eigenvectors of the variance standardized G-matrix and the genetic correlation matrix, respectively). Vector loadings of G_{max} (above) and R_{max} (below) are given to the left, and vector correlations indicating similarity among G_{max} or R_{max} across latitudes and photoperiod treatments are given to the right. The diagonal elements in bold font to the right give the eigen-value of G_{max} and R_{max}, respectively, for each of the four latitude:photoperiod combinations.

Origin	Photoperiod	Group	G_{max}/R_{max} Loadings				Vector Correlations			
Variance Standardized G-matrix (G)			DEV	SIZE	WING	MASS	N_N	N_S	S_N	S_S
North	Northern (long)	N_N	0.88	0.21	0.18	0.38	**0.73**	0.84	0.96	0.23
North	Southern (short)	N_S	0.99	-0.09	0.001	-0.04	0.84	**0.78**	0.89	0.32
South	Northern (long)	S_N	0.91	0.16	0.34	0.17	0.96	0.89	**0.89**	0.13
South	Southern (short)	S_S	0.25	-0.57	-0.44	-0.64	0.23	0.32	0.13	**0.95**
Genetic Correlation Matrix (R)			DEV	SIZE	WING	MASS	N_N	N_S	S_N	S_S
North	Northern (long)	N_N	0.36	0.49	0.54	0.58	**2.74**	0.89	0.99	0.87
North	Southern (short)	N_S	-0.10	0.53	0.57	0.62	0.89	**2.09**	0.86	1
South	Northern (long)	S_N	0.42	0.56	0.47	0.54	0.99	0.86	**2.81**	0.84
South	Southern (short)	S_S	-0.14	0.59	0.56	0.56	0.87	1	0.84	**2.65**
